# Are the Chinese Saving for Old Age? The Relationship between Future Pension Benefits of 45–60 Years Old Chinese and Current Household Expenditures

**DOI:** 10.1007/s12062-016-9159-x

**Published:** 2016-10-07

**Authors:** C.E. van Dullemen, I. Nagel, J.M.G de Bruijn

**Affiliations:** Free University, Amsterdam, Netherlands

**Keywords:** China, Ageing, Household savings, Dependency-rate, Pension coverage, Economic growth

## Abstract

Worldwide, older people’s support used to be the adult children’s responsibility. In China, two generations after introducing the one-child policy in the late 70-ies, this becomes an increasingly demanding obligation. The Chinese government took the responsibility to mitigating old- age poverty risks and realized unprecedented progress in pension coverage. At the same time, the household savings increased to about 30 % of disposable income. Built on previous research on the politics of ageing, this study analyses households responses to the established governmental and firm pension programs as well as to the New Rural Pension Scheme (NRPS), introduced in 2009. The central question is: will participation in the established and new pension programs lead to higher current Chinese household expenditures and therefore to lower savings? The China Health and Retirement Longitudinal Study (CHARLS) dataset of 2011 offered the opportunity to study the influence of the recently introduced NRPS. We find that Chinese households with members between 45 and 60 years who expect future benefits of NRPS do not have higher expenditures than those not covered by NRPS. For the participants in the established, mostly urban pension programs a correlation was found with higher current expenditures (28 % more spending on basic needs, 80 % more on luxury) However, further analysis shows that this correlation cannot be interpreted as a causal relationship. This implies that coverage by pensions, be it in urban or rural programs, does not determine higher current expenditures and lower savings.

## Introduction

Over the past two centuries, the family care system changed in a wider geographical context. Also in Asia, demographic changes, declining birthrates, changing gender roles, urbanization, and the HIV/aids pandemic are the main drivers of change of the family care system (Binstock et al. 2006, Schaie and Uhlenberg 2007; Jackson et al. 2008). At the same time, the quality of living in China is steadily advancing, poverty has declined, the average life expectancy has increased from less than 60 years in 1990 to almost 75 years today (CIA Factbook 2012). Even more remarkable is the rise of the average life-expectancy *after* 60: from 10 to 19 years.^1^ The average retirement age in China today is 60 for men and 50–55 years for women. These demographic, as well as the economic changes, have been heavily influenced by the one-child policy. What are the implications for the wellbeing of (future) older people if support can no longer be fully provided by the family?

In the last decades, the Chinese government gradually took up the responsibility to mitigate the risk of old age poverty. From the nineties onwards, the pension system was developed from pension programs for then mainly state enterprise employees and the reform of the Basic Old Age Insurance Scheme (BOISE), to the introduction of a New Rural Pension Scheme (NRPS - see annex 1) in 2009 (Shen and Williamson 2010, Quan 2012; Li 2016, Liu and Sun 2016). For the first time, compulsory coverage quotas for both urban and rural systems were included. Since its introduction, 89 million people have started receiving pension payments under the NRPS. Combined with those receiving payments under earlier established pension schemes,^2^ this means that 60 % of those aged 60 plus, received a monthly pension in 2013.^3^ Will this income security encourage the Chinese to spend more of their current income and therefore start saving less?

Until today, the household savings rate is 30 % of disposable income, one of the highest saving rate in the world after India. Why the Chinese save so much is a central issue in the debate on global imbalances and is not yet fully understood (Cristadoro and Marconi 2010). This brought up the research question for this paper: will participation in the established and new pension programs lead to higher current Chinese household expenditures and therefore to lower savings?

Based on theories and earlier research, we analyzed a dataset collected in 2011 by the researchers of the China Health and Retirement Longitudinal Study (CHARLS) to estimate the impact of pension coverage on the current expenditure rates of “middle-aged” households. The empirical literature on household savings in China is quite ample. It consists mostly of aggregated data analysis and shows a positive relationship between income and household savings. Very few studies on savings include pensions as a form of future income security. The contribution of our research is that we use a research design on *household level* instead of *aggregated level.* Furthermore, we compare different pension programs as well as current expenditures of households in urban and rural areas in China.

The paper is structured as follows: second section addresses the contextual factors like the demographic and socio-economic changes and other determinants that contributed to the explanation of Chinese high saving rates since the last 25 years. Third section presents the data, method, and variable construction, as well as the research design and results. In fourth section we present our conclusions and discussion.

## Contextual Factors

### China’s Socio-Economic and Demographic Developments

In the economic literature, savings are often conceptualized as household income minus household expenditures. In this research, household expenditures are taken as a proxy for savings. Not much research is conducted to the effects of pension programs on the current expenditures/savings of “middle-aged” households (with members of 45–60 years). Before we present our empirical findings, we first analyze the various household saving motives presented in the existing literature. Loayza et al. (2000) review the current state of knowledge on the determinants of saving rates based on a research project “Saving Across the World.” This review identifies the *non-policy determinants of saving*. These include *persistence, income, growth, demographics*, and *uncertainty*. We use these determinants as a classification and apply them to the Chinese context.

### Persistence – the gradually Changing Household Saving Rates

According to Loayza et al., household saving rates tend to show *persistency* or inertia; that is, they are highly serially correlated (Loayza et al. 2000). How did the saving rate in China change over time? From 1958 to 1975, the average Chinese household saving rate was quite low, around 5.3 % (Gomel et al. 2013). In this period, the great famine killed at least an estimated 30 million Chinese as a result of new policy and bad weather conditions that devastated the harvests (Meng et al. 2015). Under the Communist plan economy, basic housing, healthcare, and pensions were provided to workers in state enterprises, in rural areas nothing. In the seventies, the savings rate gradually increased to around 11 % in 1980. After the 1990s, this social system included in the lifelong job security for party members (“iron rice bowl”) was reformed. The state-market economy was introduced, new social security systems were set up by the state as well as commercial enterprises. The household savings kept rising steadily further to over 30 % in 2009. These savings are partly destined for old age (Hung et al. 2010; Mees and Ahmed 2012).

Also on an aggregate level, Horioka and Terada-Hagiwara analyze the determinants of the domestic saving rate in developing Asia during 1966–2007. They find that the main determinants appear to be the aged dependency ratio, income levels, and level of financial development. They project future trends in domestic saving rates in developing Asia for 2011–2030 and, based on their estimations, they predict the saving rates in developing Asia as a whole will stay steady, at least for the next two decades.

### Income and Growth

The far-reaching economic reforms from the eighties onwards, as well as the one-child policy, have fueled a remarkable economic growth, an increase in per capita income and an unprecedented decline in the poverty rate. In three decades, from 1981 onwards, the proportion of the population living in poverty in China fell from 53 % (World Bank 2016)^4^ to about 6 % in 2011.

To explain why households kept saving despite rapid income growth, IMF-economists Chamon et al. (2008) use household-level data from statistical yearbooks. They find that the saving curve is U-shaped: households with relatively young members as well as households with relatively older member save more than “middle-aged” households. The economists conclude that these high savings are related to the expected expenditures on housing, education (for younger households), and healthcare (for older households).

### Demographics

**Fig. 1:**
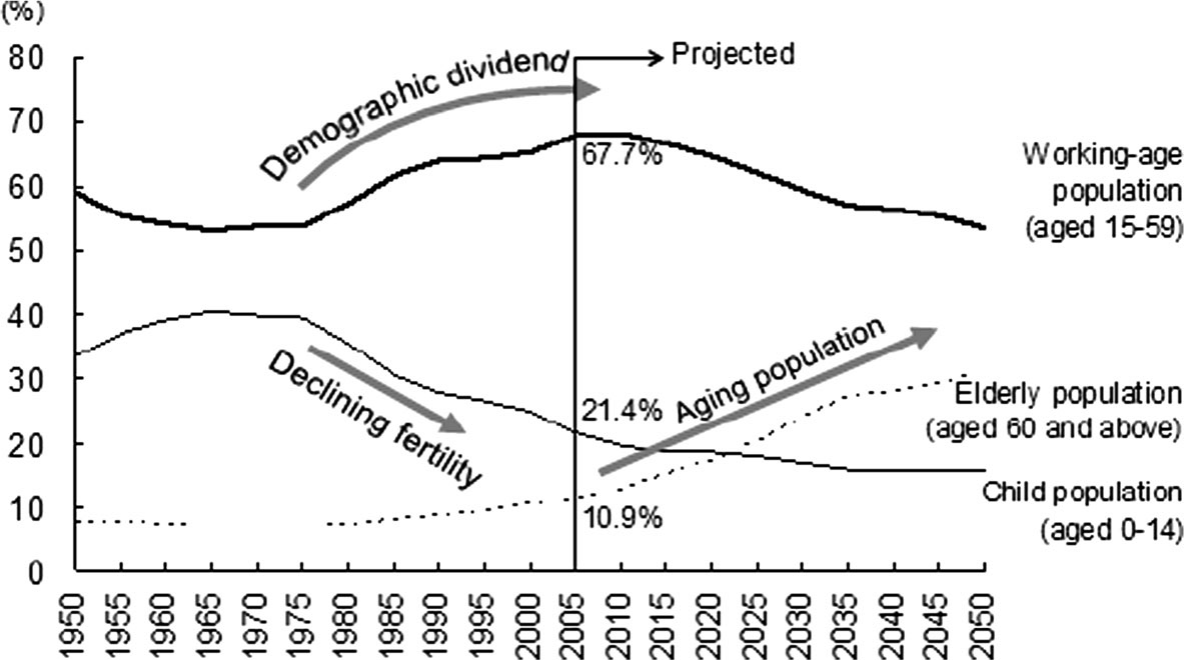
Changes in China’s population age composition

The rapid demographic changes, due to the one-child policy, lead to an increasing dependency ratio. This ratio refers to the number of younger and older dependents per total wage earners.^5^ It relates to the so-called demographic dividend (Fig. 1), to the economic growth potential that results from the transition of a population’s changing age structure. This demographic dividend is supposed to be a driving factor that fuels the economic growth which led to higher incomes as well as higher saving rates.(Chamon et al. 2010). Figure 1. shows the demographic dividend built up from the 1970-ies to 2005 while birthrates were decreasing. This demographic transition, the change from high birth and death rates to lower birth and death rates, coincide with China developing from a pre-industrial to a more industrialized country.

In their quantitative analysis of Chinese household savings Curtis et al. (2011) show that birth rates are inversely related to a country’s savings potential: having only one or two children gives the possibility to save more. Other researchers like Horioka and Wan (2006, 2007), Hung et al. (2010) see China’s still relatively low old age dependency ratio as predominant driver of its high saving rate, and further, to a lesser extent, its economic growth rate and weak social safety nets as well as educational levels.

Demography and growth are also the main determinants of aggregate saving in the research of Modigliani and Cao (1996). They tested the life cycle perspective (younger and older people spend more and middle-aged people save more) with Chinese aggregate data spanning almost 50 years (1953–2000), concluding that the theory fits the data well (Modigliani and Cao 1996). Their evidence has been questioned by research based on provincial level data by Horioka and Wan (2006) and household level data by Zheng et al. (2009), Chamon and Prasad (2010) and Brugiavini et al. (2013).

Demography and income security are also related to son preference, often related to greater anticipated old-age support from sons than from daughters and the absence of formal financial mechanisms for families to save for retirement. Ebenstein & Leung uses the introduction of a voluntary old-age pension program in rural China in the 1990s to show that parents with sons are less likely to participate in pension plans and that providing access to pension plans affects parental sex-selection decisions. Wei et al. (2011) identify Chinese parents with a son, who raise their savings in order to improve their son’s relative attractiveness for marriage. According to the researchers, this factor could potentially account for about half the actual increase in the household savings rate during 1990–2007.

The old-age dependency was one of the main determinants of household savings rates besides disposable income in the research of Mees and Ahmed (2012). They use a dataset that covers 5 decades of savings rates in China (1960–2009) and show that the main determinants of China’s household savings rates are disposable income and the old-age dependency rate. The (decreased) young age dependency rate and the average economic growth rate played only a limited role. Their results indicate that the Chinese are saving less for their children’s education than for their own expected health and old age expenses.

### Uncertainty

Theory predicts that greater uncertainty raises the saving rate since risk-averse consumers set resources aside as a precaution against possible adverse changes in income and other factors (Sandmo 1970; Skinner 1988; Zeldes 1989; Miao 2004; Loayza et al. 2000). Cristadoro and Marconi (2010, 2011) find that precautionary motives and what they identify as liquidity constraints - inability to make a purchase due to lack of cash - are the likely causes of the high household savings. They use aggregated data on disparities in saving rates between rural and urbanized provinces in China. Their results suggest that in order to reduce the propensity to save of Chinese households it is necessary to improve social services provisions and to facilitate the access to credit.

In contrast, Kraay (2000) in his empirical study on China, find that *income uncertainty* or *demographic factors* have *no* effect on savings, although he suggests that the fact that saving rates of rural households are often higher than those of urban households may partly reflect the greater uncertainty of rural incomes. Kraay uses panel data on Chinese provinces from China’s household survey to analyze the determinants of the saving rates of rural and urban households between 1978 and 83 and 1984–89. He finds that in rural areas, the expectations of *future income growth*, as well as income levels higher than subsistence consumption, play a significant role in the evolution of savings in rural areas. In urban areas, according to his findings, virtually none of the explanatory variables has a significant impact on the household saving rates.

In conclusion, the favorable low dependency rate - few children, few older people- made household expenditures and savings possible. At the same time, in the uncertainty that foreseen future holds, with high old age dependency rates and only one child to take care of older parents, households might rely on precautionary savings. In rural areas, were income uncertainty is higher than in urban areas, this counts even more.

### Policy Changes: Reforms of the Pension System

Part of the spectacular poverty reduction was due to government policy. In 1991 China’s pension system was developed for then mainly state enterprise employees and consists of three “pillars”: a public social security net (Pillar 1), a job based basic pension insurance (Pillar 2) and a supplementary private pension insurance funds (Pillar 3). In 1997 the coverage was extended to all enterprises: state-owned enterprises, collective enterprises, foreign-funded enterprises, joint-venture and private enterprises. Also, the Basic Old Age Insurance Scheme (BOISE) was reformed and covered statutory participants, employees with labour contracts (migrant workers included) but also self-employed as voluntary participants. (World Bank 2005, 2008).

In the Five Year Plan of 2005–2010, the Chinese government aimed to rebalance the skewed economic development and introduced a new pension scheme to improve the coverage of the 357 million urban residents (Lu 2012). In the Twelfth Five-Year Plan (2011–2016) *rural pensions* were prioritized.^6^ (Gao et al. 2012). With the NRPS, introduced in 2009 and cemented in 2011, the older generation is better safeguarded from old age poverty. The Chinese government has the ambition to implement a unified and standardized pension system to cover its rural and urban population before 2020 (Dorfman 2010; Dorfman et al. 2012; Zheng and Zhong 2016).

### Pensions in Relation to Savings and Expenditures

To what extent increasing government social expenditures, like this standardized pension system, can make a substantive contribution to increasing the household expenditures in China and therefore diminish savings? This is also the interest of Baldacci et al. (2010). They find that a sustained 1 % of GDP increase in public expenditures, distributed equally between education, health and pensions results in an increase of the household consumption rate of 1,25 percentage points of GDP (Baldacci et al. 2010). This outcome is interesting with respect to our research question since the NRPS is a matching program (individuals have to contribute themselves) and therefore a government social expenditure.

With respect to the direct correlation between *pension reform and household savings*, Feng, He and Sato focused on the high household saving rates in *urban China*. Their outcomes show that the pension reform, which was *less* generous for *future pensioners*, *boosted* the household saving rate *up* by about 6 percentage points for cohort aged 25–29 and by about 3 percentage points for cohort aged 50–59. Their results also indicate that declining pension wealth reduces expenditure on education and health more than on other expenditures items, in order to save more (Feng et al. 2009).

The study of Wang (2014) uses the CHARLS pilot wave of 2008.^7^ He compares urban and rural areas and different types of endowment insurance (= pension) and uses the household expenditures as a proxy for savings. He finds that the Chinese partially funded pension system stimulates consumption and squeezes out the savings to some extent which differs for the various pension types: when a pension increases 1 yuan, the growth rate of average monthly consumption will rise up by about 0.3 %–0.6 %. However, to what extent this effect is actually *caused* by the pension coverage is a question.

In the next paragraph we describe the data, the research design and the results.

## Data, Method, and Variable Construction

### Data and Research Design

The survey of the China Health and Retirement Longitudinal Study (CHARLS)^,^ 8 presided over by the Peking University, is based on a nationally representative sample of households with members aged 45 years or above (see also Zhao 2009). The purpose of CHARLS is to study the main health and economic adjustments to rapid population ageing in China.

CHARLS is based on a multistage probability sample in which first counties were sampled (except Tibet) then villages or neighborhoods, and, next, households within these. Only households, in which a member was 39 or older, were eligible as the main respondent (if the eldest person was between 39 and 45, he or she was not interviewed but was sampled for a future round of CHARLS). If present, also the spouse of the main respondent, was selected for participation in the research. In total 17,708 individuals from 10,069 households in 450 communities participated in the research. The face-to-face interviews were held between June 2011 and March 2012 at the respondents’ home by trained interviewers using CAPI technology. Information on the household income, expenditures and collectively held assets is provided by the household’s financial respondent who is most informed on these issues. Community-level information was collected by county-level interviews with a representative from the village committee or community office.

As the aim of this study is to estimate the effect of *future* benefits from pension on *current* household expenditures, we only include households in which the main respondents and their spouses are both below the age of retirement,^9^ have not retired early, and do not currently receive a pension. We also left out 198 households where information was available from only one of the spouses in a married couple. After selection of respondents in households that apply to the selection criteria and some remaining missing values in the variables of interest, we included 2330 households in our final analyses, nested within (after selection) 422 communities (within counties). We correct standard errors for this clustering in our analyses, that are conducted in Stata 13.0 (StataCorp 2013).

In the cross-sectional research design of the CHARLS survey (Zhao and Xu 2002; Zhao et al. 2009), both participation in a pension program and current expenditures may be outcomes of the same processes. OLS regression coefficients, therefore, cannot be interpreted as causal effects. To deal with this problem of endogeneity we use instrumental variable regression to estimate the causal effect of participation in these pension programs in a more stringent way (Angrist and Pischke 2008). In the next section, we first describe the construction of the variables of interest (see Table 1), and then return to the choice of instrumental variables that are used to estimate causal effects.

**Table 1:** Descriptive information dependent and independent variables

	Min	Max	mean	Stddev
Households (*N* = 2330)				
Monthly expenditures on necessities (in 100 yuan)	.01	260	14.26	14.25
Monthly expenditures on luxury (in 100 yuan)	.01	235	7.38	14.31
Participation in pension program of government and/or firm ^a^	.00	1.00	.08	.24
Participation in pension program of NRPS ^a^	.00	1.00	.24	.41
Participation in private pension program ^a^	.00	1.00	.08	.25
Expected monthly amount of firm/government pension (centered) (in 100 yuan) ^b^	-.02	113	-.0	9.25
Expected monthly amount of private pension (centered)(in 100 yuan)	-.0	43	0	2.17
Expected monthly amount of NRSP (centered) (in 100 yuan)	-.0	9	0	.60
Income from salary of main respondent and spouse (in 100 yuan) ^b^	.00	426	13.22	21.30
Income from other sources of main respondent and spouse(in 100 yuan) ^b^	-167	67	-.13	5.52
Income from subsidies of main respondent and spouse(in 100 yuan) ^b^	.00	58	.13	1.66
Income other household members and from household capital (in 100 yuan)	-.67	276	14.22	21.72
Level of education ^a^	.00	0.88	.29	.15
Age/10 (45 = 0) ^a^	.05	1.40	.54	.29
Agricultural hukou status ^a^	.00	1.00	.91	.26
Household size minus 1	.00	11.00	2.76	1.59
Communities (N = 422)				
Pricelevel (percentile ranking 0–1)	.00	1.00	.49	.29
Modernity (percentile ranking 0–1)	.01	1.00	.49	.29
Urban: rural village or urban community	.00	1.00	.29	.44
Year of introduction NRPS 2008	.00	1.00	.13	.33
Year of introduction NRPS 2009	.00	1.00	.17	.37
Year of introduction NRPS 2010	.00	1.00	.12	.33
Year of introduction NRPS 2011	.00	1.00	.09	.29
Year of introduction NRPS 2012	.00	1.00	.47	.50

Source: CHARLS 2011

^a^average of main respondent and spouse

^b^sum of main respondent and spouse

## Construction of Variables^10^

### Dependent Variables

#### Household Expenditures

For the analysis, we differentiated between household expenditures to basic needs and to luxury goods. The idea is that luxury goods, more than necessities, will be influenced by decisions to save or to spend money. As basic needs we considered food, utilities (water and electricity), fuel (including gas, coal), local transportation, toiletries (soap, toothpaste), heating, medical expenditures, purchase, maintenance and repair of transportation vehicles and communication products, taxes and fees turned over to the government and property management fees (parking). As expenditures on luxury goods we defined the costs of eating out, alcohol and tobacco, communication fees (telephone, internet use), fees for servants, culture (books, newspapers, DVD’s, cinema, bars), clothing and bedding, long distance travelling, furniture and durable goods (refrigerator, tv), education and training, fitness expenditures, beauty (facials, massage), automobiles, electronics, and donations to society. All items were added up to arrive at the two measures of household expenditures. Finally, we took the natural logarithm of both measures of expenditures to correct for the skewness in these measures.^11^

### Independent Variables

#### Participation in a Pension Program and Expected Amount of Pension

Respondents who ever worked and did not process retirement (yet) were asked if they were enrolled in a pension program of the government and institutions or of the firms. The answer defines the two pension variants: government pension and firm pension, both assumed to be part of the Basic Old Age Insurance Scheme (BOISE), aimed to cover all urban employees (Quan 2012). Subsequently, participants were asked for the expected amount of pension, in yuan per month or as a percentage of the pay at retirement (as the latter was not asked) we took the percentage of current wage from employment). For those who indicated that their participation in the pension program was too short to receive a pension and they would therefore get a one-time payment at retirement instead of pension, the amount was set at the median of those in the same situation but who would pay extra premiums. For those with a future government or firm pension the average pension benefits are estimated at monthly 1487 yuan, monthly.

All respondents, regardless if they ever worked, were asked whether they were enrolled in one of the following ‘private’ pension programs: supplemental pension insurance of the firm, commercial pension, rural pension, residents’ pension, urban residents’ pension, or another pension. In the analyses we consider all these pensions as private pensions. Respondents that had a commercial pension reported the expected amount of pension per year, per month, or, if this applied to them, as a lump sum. If the respondent had a rural, residents’ or urban residents’ pension, or another kind of pension, the expected amount could be given in yuan per month or, if that applied, as a lump sum. If there was a lump sum, it was divided by 139 months, as an estimate of remaining years after retirement. The very few respondents that had a supplemental pension insurance of the firm (.1 %) all reported the expected amount of pension in yuan per month. The estimated amount of pension from a private pension program is on average 256 yuan.

If the respondent said to participate in the New Rural Social Pension Insurance program, it was first established since when he or she (maximum 4 years) participated and how long it would take to age 60 (men) or 52.5 (women retire between 50 and 55 years). The number of participation years was then multiplied by the annual payment and the factor 1.3, to get a measure of the contribution to the pension program. If the contribution was given as a lump sum amount, this amount was multiplied by the factor 1.3. The pension amount for the New Rural Social Pension Insurance program was constructed as 55 plus the contribution divided by 139 (the expected number of months after retirement). The pension benefits of the NRPS pension program are on average 70 yuan per month. 2.0 % of the respondents reports participating in more than one pension program. The expected amount of pension is expressed in 100 yuan.

To attain variables at the household level, we took the average of the main respondents’ participation in each pension program. Therefore, household participation in a pension program can be 0, .5 or 1. To arrive at the household’s expected amount of pension we summed the expected pension amount of the main respondents.

### Control Variables

Following Wang (2014) we analyze both household expenditures while controlling income, which is expected to be the main determinant of expenditures. Moreover, current saving, which is the main interest of this study, is defined by expenditures minus income. Apart from income we include some other variables that may explain some variation in household expenditures, and to ensure that any direct effects of the instrumental variables are captured by the controls. Moreover, the effects of the control variables give an indication of the validity of the dependent variables. Next to income, we control the price level of the community, income, education, age (as in Wang 2014),^12^ and also family size, the level of modernity and the degree of urban-rural of the community.

### Income

Individual incomes of the main respondent and the spouse were asked at two points in the questionnaire. In the section on household income respondents reported the amount of wage and bonus income in the past year. From the information in the module on work retirement a total measure of monthly wage income is constructed as the sum of (self)employment, farming and unpaid family business. We took the average of two measures of individual income as a measure of individual *income from salary* (*r* = .45). Besides this measure, a separate measure of *individual income is from stocks, funds, and other investments* which can also take a negative value. A third measure of *income is from subsidies* or individual-based transfers: pensions, unemployment compensation, pension subsidy, worker’s compensation from Industrial Accident Compensation Insurance, elderly family planning subsidies, medical aid, other government subsidies, social assistance, other income sources like alimony and child support. The *household income* consists of income from agriculture, self-employed activities, income from wage and subsidies of individual household members other than the main respondents, household subsidies, household income from other resources. All measures of income are expressed in the amount of 100 yuan per month.

### Household Education, Age, Size of the Household

The highest level of *education* attained varies mainly between no formal education (illiterate) (14.2 %) to some education (capable of reading or writing, elementary school) (35.7 %)) and middle or high school (46.0 %). 4.0 % has attained vocational school or college. For the measure of education of the household, we took the average of the main respondents. *Age* is based on the year of birth, and if missing, on a subsequent question that directly asked for age. In our selection of the sample, age varies between 45 and 59. For the analyses we subtracted 45, so that it is expressed as deviations from 45. Here also we took the average age of the household, and expressed it in 10 years of age. The *size of the household* apart from the main respondent and spouse was constructed as the maximum of the number of household members named in the household roster when asked for gender and birth year. The total number of persons in the household number varies between 1 and 12.

### Community Price Level, Modernity, Urban

Following Wang (2014), we control the community’s *price level* of food and other commodities, as provided by the village committee or community office (the liji part of pork, chicken eggs, rice, flour, natural gas, LPG, water, electricity, coal, a new and used apartment, and the rental price of an apartment). The measure of price level was constructed as the average of the percentile rankings of these variables (Cronbach’s alpha .75, eggs left out) and ranked it into a 0–1 range. We also include a measure of *modernity*, based on information given by the village committee or community office on the management of waste in the community, main type of toilet, availability of different schools, services, and stores. As a measure of the level of modernity we took the average of the percentile rankings of these variables (Cronbach’s alpha .80), ranked this variable again into a 0–1 range. The correlation between modernity and price level is .475. As part of the household interview the interviewer was asked to take down the type of the neighborhood, *rural village or urban community*. As a community level measure of urbanization we took de community average of these answers. Although most of the interviewers – on average 23 (between 2 and 53) per community - agree on the type of neighborhood, typifying the community as either rural or urban, in 11 % of the cases their ratings differ somewhat, in which cases the community level variables is between 0 and 1.

### Instrumental Variable Regression

We use instrumental variable regression analysis (ivregress in Stata) to deal with the endogeneity problem, i.e. that participation in the pension programs and the household expenditures could be outcomes of the same processes, or to confounding variables that are omitted from the analysis. Instrumental variables have to fulfill two assumptions. The first is that the instrument correlates reasonably well with participation in the pension program. The second assumption, the exclusion restriction, is that the instrument approaches random assignment, independent of the outcome, conditional on covariates and that instruments only affects the outcome variable via the treatment variable (Angrist and Pischke 2008, 117).

As the first set of instrumental variables^13^ to estimate the effect of participation in a pension program we use the year of introduction of the NRPS pension program. The pension program NRPS was officially introduced from 2009 onwards, not at the same rate in all provinces, first in pilot locations (Quan 2012, 14). The *year of introduction of NRPS* could serve as an instrumental variable if we assume earlier introduction will, through increased public knowledge, affect the probability of taking part in the NRPS pension program, and (only) this way will affect the current expenditures. The community’s year of introduction of the NRPS program, however, does indeed relate to participation in this pension program. As we think it is reasonable to assume that the community’s year introduction does not directly affect the household expenditures, we use this as an instrumental variable.

As community information on the introduction of NRPS is only available for 307 of the 450 communities, we created an alternative measure of the year of introduction, for which we used the respondents’ survey and took for each community the first year in which respondents (full sample) said to have started their participation in NRPS.^14^ These years vary between 2008 and 2011. For communities in which not a single respondent said to participate in NRPS, we assumed the year of introduction to be 2012. This information is available for 440 out of the 450 communities.

As an additional instrument, we use individual *HuKou status,* the Chinese household registration system. Due to policy measures of the communist regime in the previous century in which people, were forced to live in rural areas, the Hukou status – agricultural or non-agricultural – is at least partly exogenous. Residents with an urban HuKou are thought to participate more often in government and firm pension programs; for the NRPS pension, only those with an agricultural HuKou are eligible. As access to the two pension programs is also conditional residents with an urban HuKou are allowed to participate; for the NRPS pension, only those with an agricultural HuKou are eligible. As access to the two pension programs is also conditional on the Hukou status, we use HuKou status as an instrument for participation in both pension programs. More precisely, as a minority of respondents changed their Hukou status, we use their *first* HuKou status. Here we follow Lizardo (2006) who uses region of residence in childhood and ‘type of locale’ as instruments in his study on cultural taste and networks size (795). Respondents were asked to report their first Hukou status, that varies between agricultural (91.3 %) and non-agricultural (8.7 %) HuKou.^15^

Table 2 presents the first stage instrumental regression results: the determinants of participation in the two pension programs that are instrumented, the government or firm pension program for urban employees, and the NRPS program, for rural residents. These first stage regression results are interesting in itself, but also serve to test the first assumption that the instrumental variables significantly affect participation in these pension programs.

**Table 2:** First stage 2 sls regression of participation in pension program of government and/or firm and NRPS pension, OLS regression coefficients and t-values based on robust standard errors (N households =2330, N communities =422)

	Pension government / firm		NRPS pension	
	B	t	B	t
Private pension	-0.0134	-0.8	-0.3037	-7.1*
Income salary	0.0018	3.6*	0.0000	-0.1
Income other	-0.0008	-0.9	0.0007	0.8
Income subsidy	0.0016	0.5	-0.0007	-0.5
Income household	-0.0005	-2.0*	0.0005	1.5
Age	-0.0124	-0.9	0.0379	1.4
Education	0.3333	7.5*	0.0958	1.4
Household size	0.0009	0.2	0.0049	0.9
Price level	0.0314	1.4	-0.1244	-2.2*
Modernity	0.0187	0.7	-0.0566	-0.9
Urban	0.0681	2.4*	0.0106	0.2
Community introduction NRPS 2009	0.0199	0.9	0.1033	1.8
Community introduction NRPS 2010	-0.0068	-0.3	0.1898	3.2*
Community introduction NRPS 2011	0.0060	0.3	-0.0087	-0.1
Community introduction NRPS 2012	0.0162	0.8	-0.3457	-8.4*
Hukou agricultural	-0.1805	-4.2*	0.0980	2.6
Cons	0.0942	1.7	0.2791	4.4*
F (18, 2311)		10.37*		25.16
R-squared		.26		.30

*p < .05

The first column of Table 2 shows that households with (one or two) government or firm pensions tend to have a higher average income from salary, are higher educated, live in urban regions, and have lower household income, and, have less often an agricultural Hukou status. People who are enrolled in NRPS live in less “expensive communities”.^16^ Participation in NRPS negatively relates to participation in private pension program. Households with (one or two) agricultural Hukou’s indeed are more likely to participate in the NRPS pension program. Also, and as expected, the year of introduction of NRPS in the community, does affect participation rates in NRPS, though not linearly, as was expected. With respect to the first assumption, both Hukou status and year of introduction of NRPS are valid instruments.

Although we cannot really test the second assumption that the instruments affect the expenditures only via participation in the pension program, we approach such a test by an OLS regression of expenditures on the instrumental variables and control variables, following Lizardo (2006). The results (in Tables 3 and 4) show that the relation between expenditures and the year of introduction of NRPS is not significant, given the control variables. The same holds for the effect of Hukou status on expenditures on necessities. However, the effect of Hukou-status on the household expenditures on luxury is significantly positive, which points to a violation of the exclusion restriction. However, if we would exclude the Hukou status as an instrumental variable, in particular participation in the pension program of government and firm is very poorly predicted by the available instruments, which is not in line with the first assumption again. We decided to leave Hukou status as an instrument in the analyses, and, as the effects of participation in the pension programs cannot fully be interpreted as causal, we will interpret the results with caution.

**Table 3:** Household expenditures (natural logarithm) on necessities and participation in pension program of government and/or firm and NRPS pension, OLS and 2SLS regression coefficients (instruments: year of community introduction NRPS, agricultural Hukou status), z-values based on robust standard errors (N households =2330, N communities =422)

	1 OLS		2 OLS		3 2SLS	
	b	t	b	t	B	z
Pension government / firm	0.2437	3.6*			-0.2534	-0.6
Pension NRPS	0.0209	0.4			0.0872	0.8
Private pension	0.0971	1.3			0.1072	1.3
Amount of pension government / firm			0.0042	2.2*		
Amount of pension NRPS			0.0295	0.9		
Amount of private pension			0.0199	3.9*		
Income salary	0.0034	3.3*	0.0034	3.1*	0.0043	3.1*
Income other	-0.0025	-1.5	-0.0021	-1.2	-0.0030	-1.6
Income subsidy	0.0021	0.1	0.0027	0.2	0.0029	0.2
Income household	0.0001	0.2	0.0001	0.2	-0.0002	-0.2
Age	-0.2305	-3.9*	-0.2342	-3.9*	-0.2389	-4.0
Education	0.5053	3.4*	0.5162	3.4*	0.6611	2.9
Household size	0.1346	10.4*	0.1341	10.2*	0.1343	10.3*
Price level	0.2639	2.8*	0.2680	2.9*	0.2927	3.2*
Modernity	0.2738	2.6*	0.2693	2.6*	0.2844	2.8*
Urban	0.1330	1.9	0.1292	1.9	0.1580	1.9
Introduction NRPS 2008	0		0			
Introduction NRPS 2009	0.0316	0.4	0.0395	0.5		
Introduction NRPS 2010	0.0131	0.2	0.0139	0.2		
Introduction NRPS 2011	0.0372	0.4	0.0407	0.4		
Introduction NRPS 2012	-0.0144	-0.2	-0.0122	-0.2		
Hukou agricultural	0.1086	1.4	0.0874	1.1		
Cons	1.4956	11.5	1.5456	11.9*	1.5543	16.5
R-squared		.15		.15		
Wald chi2(13)						284.3

*p < .05

**Table 4:** Household expenditures (natural logarithm) on luxury and participation in pension program of government and/or firm and NRPS pension, OLS and 2 SLS regression coefficients (instruments: year of community introduction NRPS, agricultural Hukou status), z-values based on robust standard errors (N households =2330, N communities =422)

	1 OLS		2 OLS		3 2SLS	
	b	t	b	T	b	z
Pension government / firm	0.5871	4.4*			-2.0425	-2.1*
Pension NRPS	0.0780	1.0			0.1817	1.1
Private pension	0.1280	1.1			0.1556	1.0
Amount of pension government / firm			0.0156	5.2*		
Amount of pension NRPS			0.1346	2.7*		
Amount of private pension			0.0275	2.5*		
Income salary	0.0087	6.4*	0.0080	6.2*	0.0133	4.8*
Income other	-0.0081	-1.6	-0.0063	-1.3	-0.0093	-1.6
Income subsidy	-0.0008	-0.1	0.0005	0.0	0.0032	0.4
Income household	0.0087	7.0*	0.0088	7.1*	0.0073	5.2*
Age	-0.3233	-3.9*	-0.3281	-4.0*	-0.3660	-4.1*
Education	0.7695	3.7*	0.7203	3.5*	1.6774	3.9*
Household size	0.1342	6.9*	0.1320	6.8*	0.1350	6.3*
Price level	0.3270	2.5*	0.3392	2.7*	0.3991	2.9*
Modernity	0.3718	2.6*	0.3675	2.6*	0.4058	2.6*
Urban	-0.2003	-1.8	-0.2101	-1.9	-0.0323	-0.2
Introduction NRPS 2008	0.1381	1.3	0.1447	1.3		
Introduction NRPS 2008	0.1573	1.3	0.1484	1.3		
Introduction NRPS 2008	-0.1600	-1.2	-0.1503	-1.1		
Introduction NRPS 2008	0.0225	0.2	0.0463	0.5		
Hukou agricultural	0.5422	3.7*	0.5197	3.6*		
Cons	-0.3084	-1.4	-0.1952	-0.9	0.0514	0.4
R-squared		.17		.17		
Wald chi2(13)						252.6

*p < .05

We also estimate the effects of participation in private pension programs and of the amount of pension. However, as we could not find any variables to instrument these effects, also with respect to these, we will be cautious in interpreting the effects as causal.

## Results

Table 3 presents the effects of participation in pension programs on monthly household expenditures to necessities. In the first two models, the OLS estimates, in the third the estimates from the instrumental variable regression. The results for the OLS regression reveal that there is a positive association between participation in a government or firm pension program and current household expenditures on necessities. Those with a government pension spend 28 % ((exp(.2437)) more on food, electricity, medical expenditures and other basic needs than those do not participate in such programs. Participation in NRPS or in a private pension program is not related to more expenditures on necessities.

In the second model we examine the future expected amount of pension instead of participation in the pension programs. Again, positive relations are found between the amount of government or firm pension or a private pension, whereas no differences in expenditures by the amount of expected NRPS pension occur.

In the third column of Table 3 we estimate the causal relation between participation in a pension program and expenditures on necessities. These results show that there is no evidence of any causal effects of participation in government and firm pension programs nor of participation in the NRPS program on the expenditures on necessities. In comparison with the OLS estimates in model 1, there are no significant effects of participation in government and firm pension anymore. This means that differences in expenditures on necessities between households that do participate in pension programs of government and firm and those that do not, are not *caused* by participation in the government or firm program. This implies the groups are different on variables that are not tested, f.i. individual characteristics like risk-adversity of household members who keep resources aside as a precaution against possible adverse changes in income instead of consumption, or lifestyle preferences that are more persistent and less depend on changes of income could also be an explanatory factor.

With respect to the effects of NRPS, the results lead to the same results as are obtained from the OLS regression. There are no differences between households that do and do not participate in these programs and these do not turn up when only systematic variations according to the instrumental variables examined.

There are no effects of private pension on expenditures either. Here we cannot draw conclusions on the causality of the effect, but as neither the OLS nor the IV regression points at any differences between households that take part in these programs and those that do not, there is not much evidence for a causal effect either.

If we take a look at the relations between expenditures on necessities and other characteristics, we find, as expected, that expenditures vary with the income from the salary of the main respondents in the household. Households of a larger size spend more money, and also households in communities with a higher price level. As these relations are as expected, they give confidence in the validity of the expenditures measures. A bit less straightforward are the relations with age and education. Younger households spend more money and also higher educated spend more money. In modern communities, more money is spent, and also in urban communities more is spent on basic needs. There are no additional effects of Hukou status on expenditures on necessities.

In Table 4 the household expenditures on luxury are modeled. In the first two models, OLS estimates are presented, in the last column the results of the IV regression. Model 1 makes clear that there is a positive relation between the household’s *participation* in a pension program of firm or government and the expenditures on luxury. Those with a government pension spend 80 % ((exp(.5871)) more on luxury than those who do not participate in this program. Household expenditures on luxury do *not* vary with participation in NRPS or in a private pension program. This might be due to the low amount. The maximum respondents could contribute to NRPS are men of 45 years old who would pay contribution for 15 years.

The second model, in which the *amount of pension* is included, shows positive relations between the amount of pension, of all types of pension, and the expenditures on luxury. The larger the amount of pension that is expected, the more households spend on luxury. However, these analyses do not answer the question to what extent the participation in a pension program *causes* the amount of expenditures on luxury.

The third model estimates the *causal* effects of taking part in the pension programs of government or firm and of NRPS. The positive effect of participating in a government or firm pension has disappeared now if only the exogenous variations in the instrumental variables are modeled. The effect has even become negative, meaning that participation in the pension program would lead to spending less on luxury, which is the opposite of what was expected. As there was a direct effect of the Hukou status on the household expenditures on luxury (in model 1), we are a bit careful with drawing firm conclusions. However, it seems justified to conclude that participation in this pension program does not cause an increase in the household expenditures on luxury goods. Participation in NRPS or in a private pension program does not affect expenditures on luxury either. These results are the same as in model 1.

Expenditures on luxury goods also vary with income, also from other household members, household size, age, education and are higher in modern communities and those with a high price level. Unexpectedly, households with an agricultural Hukou spend more on luxury as compared to those with a non-agricultural Hukou, but that are similar in income, education, age, pension participation and the other characteristics that were held constant. Though taking a closer look at the content, it becomes an understandable outcome. Rural inhabitants will spend more on communication fees (telephone, internet use), long distance traveling and probably also spend more on donations to society.

## Conclusion and Discussion

In China, over the past three decades, demographic trends have converged strongly with economic growth as the birthrates dropped and the aged population was not yet that sizeable. This so-called demographic dividend has contributed to the rapid socio-economic development. At the same time, Chinese household savings are with about 30 %, one of the world’s highest savings rates. Now China is approaching a tipping point. In the next three decades, demographic trends will lead to an increase of older people to an expected third of the population in 2050. The old-age dependency burden will gradually rise; tripling today’s level by 2030 and the total dependency will double.

The Chinese government realizes this challenge and is steadily working on fulfilling its promise of full pension coverage, which is particularly important in the rural areas. Pension policies could be expected to have a correlation with household expenditures and savings as we analyzed in this paper. Our central question was: will participation in the established and new pension programs lead to higher current Chinese household expenditures and therefore lower savings?

In the literature, we found consensus over the following factors having a major impact on household savings in China, including persistence, income and expected growth, demographics, and uncertainty. Research done by Feng et al. (2009), Mees and Ahmed (2012), Cristadoro and Marconi (2010, 2011) and Curtis et al. (2011) all shows that the *demographic transition* explains a significant amount of the variation in the household saving rate in China.

By analyzing the CHARLS 2011 database, we found empirical evidence for a *positive* relationship between participation in established pensions programs and monthly household expenditures. However, after introducing a more stringent test on causality, we did not find evidence that this is a causal relationship.

We have partly built on the work of Wang Zhenhui who analyzed the pilot-wave of the CHARLS dataset. He found that if the pension increases 1 yuan, the growth rate of average monthly consumption will rise up by about 0.3 %- 0.6 %. The differences with our findings can be explained by the fact that Wang used the data of the pilot study, which was not a nationally representative sample as was the wave of 2011. Furthermore, he included retired respondents who already received a pension and the fact he did not apply an instrumental variable regression to estimate the causality of the relationship. And we used an instrumental variable regression to tackle the problem of endogeneity and to estimate the causal effect of participation in various pension programs in a more stringent way. By doing so, we did not find any difference between the consumption rate of those who expected to participate in the NRPS and those who did not. Also, the amount of the expected pension did not make a difference. Our results indicate that household expenditures of “middle-aged” households are not influenced by pension participation. This suggests that there will be *no influence* on the rural household saving rate when the Chinese government will extend the pension coverage with the ambition to reach full coverage 2020.

Though our findings can also be an indication that, in spite of being enrolled in a pension, people still save more because they do not see the current NRPS as a sound income security yet. This might be due to the low amounts or as an indication of persistency of household savings. Though earlier studies show similar results that might support this, more research is required to confirm this hypothesis.

## Discussion

The scope of our research on the relationship between future pensions benefits and current Chinese household expenditures reaches beyond their domestic borders. The high Chinese savings rate has worldwide economic implications because of its perceived contribution to the global imbalances (Cristadoro and Marconi 2010) and even to the 2008 financial crisis (Obstfeld and Rogoff 2009). Some studies find a clear correlation between participation in a pension program and the amount of savings. (Ebenstein and Leung 2010; Wang 2014). We also find this relationship; those who participate in governmental or firm pension programs save less (measured by higher household expenditures as a proxy). However, by using methodological more stringent tests like instrumental variable regression method, we do not find a causal relationship. For the rural areas, this relationship between expected future benefits and current household expenditures is not found at all. This implies that future pensions benefits are not (yet) a determinant of current more household expenditures (and thus lower savings), though we recognize that persistency could be an explanatory factor of the absence of this relationship. The trust in rural areas in more future income security by participating in the rural pension program is probably not yet augmented enough to increase current household expenditures. Repeating this research with the next waves of the CHARLS data-set could be a way of verifying and fine tuning our results. Our findings are in line with Horioka and Terada-Hagiwara (2012) who expect that the domestic saving rate in developing Asia as a whole will remain roughly constant between 2007 and 2027. They envision that rapidly ageing economies will show a sharp downturn in their domestic saving rates by 2030 because the negative impact of population ageing will dominate the positive impact of higher income levels.

Another interesting issue for future research is the gender dimension of future pension benefits and current household expenditures (c.q. savings) in China. In our analyses on household level was a step ahead compare to aggregated data analyses, but we could not analyze the gender differences within households. This could be seen as a limitation because of our broader interest in income security for the aged poor, who are predominantly women. Very few academic studies are available yet on gender differences of the Chinese household savings.[1] According to a number of studies on aggregated levels, in general, women save more than men. This is found in a study based on data from South Korea (Kim 1997; Lee and Pocock 2007), from Kenya (Anderson et al. 2003) and the USA (Hungerford and Library of Congress 2006). Seguino and Floro (2003) showed in the context of developing countries, that the higher women’s income is relative to that of men, the higher is a country’s gross domestic savings rate. However, there are also researchers who make a link to marital status as the main explanatory factor of gender differences (Zhang et al. 2010). For instance, Kureishi and Wakabayashi (2013) analyzed precautionary savings due to staying single in the presence of income uncertainty. Comparing young women who are likely to get married within 3 years, with those who are not planning to get married, the last ones have 44 % more savings for precautionary purposes, and 108 % more for retirement than the first. These researchers suggest that in facing a higher risk of income fluctuation, due to choosing to marry late or remain unmarried, young women intend to have more wealth to mitigate the income risk (of losing their job and not be compensated by income of a husband) inherent in single life. Similar results are found by Grossbard and Pereira (2010). They show that a scenario of higher marriage rates and higher divorce rates will be associated with higher savings rates. In the context of traditional gender roles, this implies higher saving rates by young men and lower saving rates by young women. In more modern countries, the opposite is the case with higher saving rates of married women relative to those of married men.

The study of Seguino, & Floro indicate that when women’s discretionary income and bargaining power increase, aggregate saving rates rise, implying a significant effect of gender on aggregate savings. These findings demonstrate the importance of understanding gender relations at the household level in planning for savings mobilization and in the formulation of financial and investment policies (Seguino and Floro 2003).

In general, women live longer than men and they run a larger old age poverty risk. This might lead to lower expenditures and higher savings than men. Would this be a motive for the Chinese government to stimulate the enrollment of more women in pensions in order to realize a lower savings rate? It would be interesting to have more in-depth knowledge about the gender differences in saving behavior, again, because the Chinese saving rate has global implications. More research would be required to analyze this gender dimension as well as its relationship with various pension programs.

## Annex 1

**Table:**

Categories	New Type of Rural Social Endowment Insurance
Year of creation	2009
Basic approach	Fully funded and pay-as-you-go
Basic principles	Guaranteed basic, wide coverage, elasticity and sustainability
Guiding ideology	Sharing the pension burden among individuals, collectivities and the state
Participation	Voluntary
Eligible population	Non-student rural residents aged 16 and older and citizens not eligible to enroll in any other old-age pension scheme
Fund sources	Individual contributions, collective subsidies and government subsidies
Yearly individual contributions	Choice between 100,200,300, 400 and 500 Yuan and above
Government payment subsidies	Yearly local government subsidies for pension contributory benefits cannot be lower than 30 Yuan per person, and they are automatically added to each individual pension account
Pension structures	Basic pension and individual account pension
Eligibility conditions	Reaching 60 years old
Basic Benefit	55 Yuan per month, and local governments can increase that basic amount at will
Individual account pension	The quotient of total amount of money in the individual account and 139
Competent authority	Ministry of Human Resources and Social Security

Sources: People’s Republic of China Social Insurance Law; Guidance to carry out the NTRSEI Pilot Project, and Guidance to Carry Out the Old-age Insurance for Urban Residents Pilot Projects

In the NRPS all rural residents aged 16 or above who are not enrolled in the urban basic pension program can participate voluntarily. The pension fund consists of two main parts: an individual premium and a government subsidy. The individual premium comprises five categories: 100, 200, 300, 400, and 500 RMB^17^ per year per person. The higher the premium, the higher the nominal payments received in the future. The government subsidy comprises two sources: one from the local government, which is required to be no less than 30 RMB per year per person, and the other from the central government, also called the basic pension benefit. When a beneficiary turns 60, he/she starts to receive a monthly benefit (1/139 of the total accumulation) from the individual account for a maximum 139 months. At the same time, he/she receives a basic pension benefit (currently 55 RMB per month).

## Footnotes

1
http://www.helpage.org/global-agewatch/population-ageing-data/life-expectancy-at-60/

http://knoema.com/atlas/China/topics/Demographics/Age/Life-expectancy-at-age-60 (checked 4/10/2016)

2 At the end of 2011 the number of people participating in BOISEE was 283.9 million, of which 215.7 million were contributors and 68.2 million were recipients. The Basic Old-age Pension System for Civil Servants (BOPSCS) covers all employees working in state-owned administrative agencies and institutions, with around 40.2 million people currently working in the civil service. (Quan 2012)

3
http://www.refworld.org/pdfid/5301df5d4.pdf

4
http://econ.worldbank.org/WBSITE/EXTERNAL/EXTDEC/EXTRESEARCH/0,contentMDK:20,634,060 ~ pagePK:64,165,401 ~ piPK:64,165,026 ~ theSitePK:469,382,00.html

5 Dependency ratio is the number of dependents divided by the number of wage-earning people per household.

6 International Social Security Association (2013), Social security coverage extension in the BRICS A comparative study on the extension of coverage in Brazil, the Russian Federation, India, China and South Africa. http://apsredes.org/wp-content/uploads/2014/03/2-BRICS-report-final.pdf (checked 11/04/2016)-

7 The pilot survey of CHARLS was conducted in two provinces (Gansu and Zhejiang) in 2008 and collected data from 48 communities/villages in 16 counties/districts.

8 For more info: *China Health and Retirement Longitudinal Study* to be found at http://charls.ccer.edu.cn/en

9 In China, the general age of retirement for men is 60; for women between 50 and 55. Though the eligible age of NRPS is 60 years for men and women equally, we selected men younger than 60; women younger than 55 to keep the average age between the comparison groups equal.

10 For the construction of the variables, we applied the following procedure to handle missing values. In general, missing values were first replaced by the mean of lower and upper boundaries in the unfolding bracket questions (if present) that were used in financial questions in order to minimize the nonresponses (Zhao et al. 2009, 3). Next, missing values were replaced by zero for those who had indicated or for whom it could be assumed that the financial information that was asked for (i.e. a certain kind of income, a subsidy, way of saving), did not apply to them. Missing values for those who had indicated that the financial information applied to them, were replaced by the median (on selected sample by age and retirement) based on the other respondents or households to whom the financial information applied. To deal with possible outliers, we recoded answers that exceeded the maximum amount to the maximum mentioned in the ‘soft checks’ for the interviewer.

11 Zero expenditures were coded as 1 yuan (to be able to compute the natural logarithm).

12 Wang (2014) additionally controls health condition and family assets.

13 We first used also the *province’s coverage rates* as provided by Quan (2012) of the two main pension programs, BOISE for urban employees and NRPS for rural residents. Unfortunately, the province’s coverage rates of these two basic programs do only very weakly correlate with the participation in these programs among the 45–59 years old in the sample, which makes them unfit as instrumental variables.

14 We took this idea from a paper on the CHARLS data which used this information as an instrument. However, these authors indicate that they do not want to be cited.

15 For the .5 % with unified residence HuKou, we took their former Hukou status.

16 These are perhaps rural communities, although the measure of urban rural does not additionally affect participation in NRPS.
